# Fatal haemorrhage and neoplastic thrombosis in a captive African lion (*Panthera leo*) with metastatic testicular sex cord–stromal tumour

**DOI:** 10.1186/s13028-017-0337-5

**Published:** 2017-10-13

**Authors:** Omar Antonio Gonzales-Viera, Angélica María Sánchez-Sarmiento, Natália Coelho Couto de Azevedo Fernandes, Juliana Mariotti Guerra, Rodrigo Albergaria Ressio, José Luiz Catão-Dias

**Affiliations:** 1 Laboratório de Patologia Comparada de Animais Selvagens, Departamento de Patologia, Faculdade de Medicina Veterinária e Zootecnia, Universidade de São Paulo, São Paulo, SP 05508270 Brazil; 2 Present Address: Department of Pathology, Microbiology and Immunology, University of California, Davis, USA; 3 Centro de Patologia, Instituto Adolfo Lutz, São Paulo, SP 01246000 Brazil

**Keywords:** Immunohistochemistry, *Panthera leo*, Sex cord–stromal gonadal tumour

## Abstract

**Background:**

The study of neoplasia in wildlife species contributes to the understanding of cancer biology, management practices, and comparative pathology. Higher frequencies of neoplasms among captive non-domestic felids have been reported most commonly in aging individuals. However, testicular tumours have rarely been reported. This report describes a metastatic testicular sex cord–stromal tumour leading to fatal haemorrhage and thrombosis in a captive African lion (*Panthera leo*).

**Case presentation:**

During necropsy of a 16-year-old male African lion, the left testicle and spermatic cord were found to be intra-abdominal (cryptorchid), semi-hard and grossly enlarged with multiple pale-yellow masses. Encapsulated haemorrhage was present in the retroperitoneum around the kidneys. Neoplastic thrombosis was found at the renal veins opening into the caudal vena cava. Metastases were observed in the lungs and mediastinal lymph nodes. Histology revealed a poorly differentiated pleomorphic neoplasm comprised of round to polygonal cells and scattered spindle cells with eosinophilic cytoplasm. An immunohistochemistry panel of inhibin-α, Ki-67, human placental alkaline phosphatase, cytokeratin AE1/AE3, cKit, vimentin and S100 was conducted. Positive cytoplasmic immunolabeling was obtained for vimentin and S100.

**Conclusions:**

The gross, microscopic and immunohistochemical findings of the neoplasm were compatible with a poorly differentiated pleomorphic sex cord–stromal tumour. Cause of death was hypovolemic shock from extensive retroperitoneal haemorrhage and neoplastic thrombosis may have contributed to the fatal outcome. To our knowledge, this is the first report of sex cord–stromal tumour in non-domestic felids.

## Background

The study of neoplasia in wildlife species contributes to the understanding of cancer biology, species management [1] and comparative pathology due to the diverse cancer presentations and complementary diagnostic techniques such as immunohistochemistry in non-domestic species. Studies of tumour prevalence in captive felids are scarce and originate from zoo collections, but suggest high frequencies of neoplasia, up to 51% [2–4]. Nevertheless, testicular tumours have rarely been reported. Those previously reported include: Sertoli cell tumour in a snow leopard (*Panthera uncia*) [5], a clouded leopard (*Neofelis nebulosa*) [6], an Amur tiger (*Panthera tigris altaica*) [7], and a jungle cat (*Felis chaus*) with multiple neoplasms [8]; and seminoma in a clouded leopard [9], snow leopards and a tiger [3, 10]. Studies indicate that older non-domestic felids are more likely to develop testicular neoplasms than juveniles as is recognized in other species. Although, testicular neoplasms are rarely seen in domestic cats, seminoma, Sertoli cell and interstitial cell tumours have been reported [11]. Testicular neoplasms are most commonly found in dogs, with interstitial cell tumours occurring mainly in mature and old animals [11]. In dogs, the three main testicular neoplasms are the two types of sex cord–stromal tumours: the Sertoli cell tumour (sustentacular) and interstitial (endocrine) Leydig cell tumour, and the germ cell tumour: seminoma. The next most common type is the mixed germ cell-sex cord stromal neoplasm (gonadoblastoma) [11]. This study describes a case of metastatic testicular sex cord–stromal tumour with fatal haemorrhage and neoplastic thrombosis in an African lion (*Panthera leo*).

## Case presentation

**Fig. 1:**
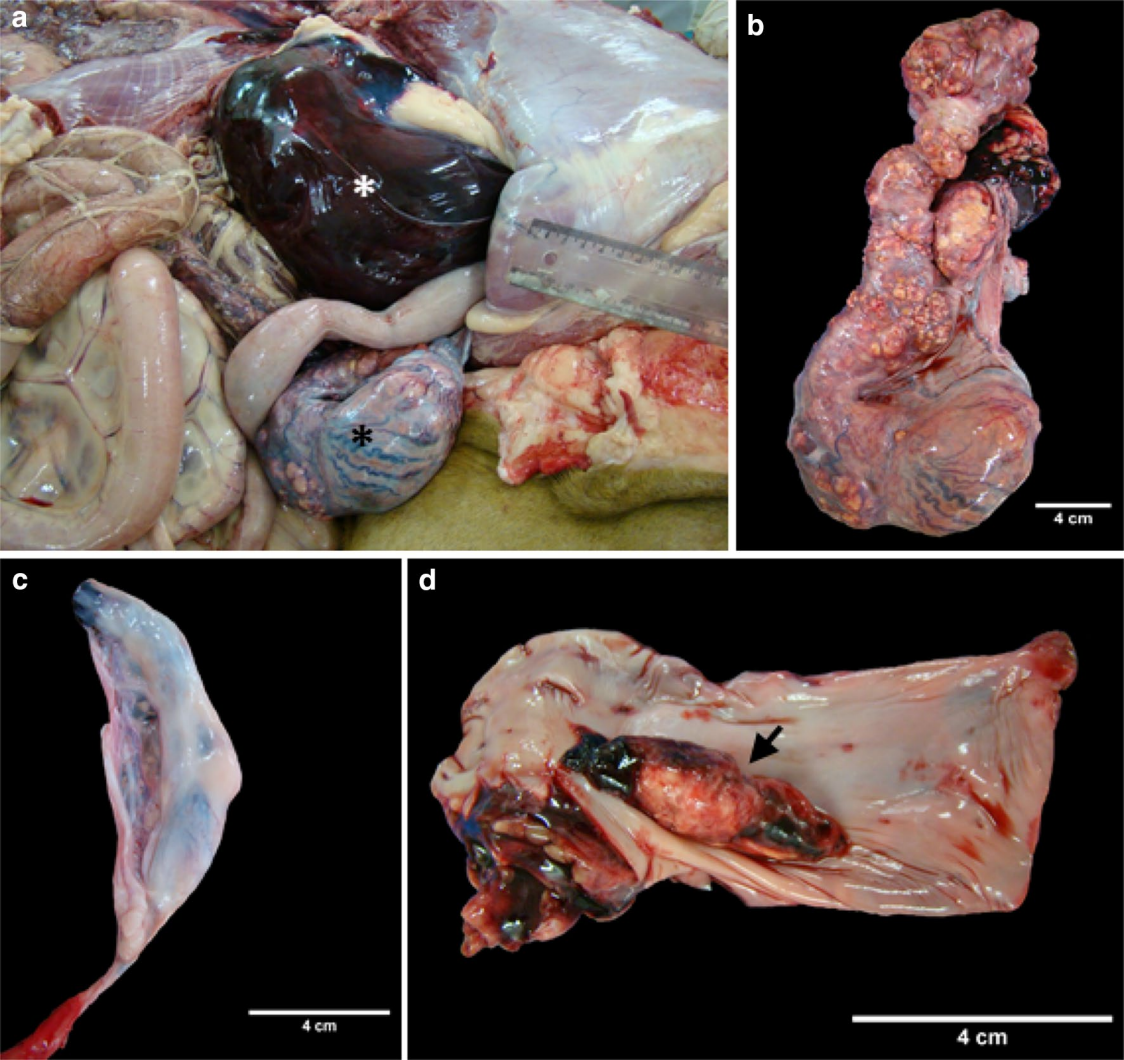
Gross images, African lion. **a** Abdominal cavity, note marked encapsulated haemorrhage on the parietal sub-peritoneum around the left kidney (white asterisk) and enlarged left testicle (black asterisk). **b** Enlarged left testicle exhibiting multiple yellow nodules involving the epididymis, pampiniform plexus, and deferent duct. **c** Right testicle evidencing decreased size. **d** Marked thrombus at the opening of the renal veins into the caudal vena cava (arrow)

A 16-year-old male African lion was presented for necropsy to the School of Veterinary Medicine and Animal Sciences, University of São Paulo, Brazil. The animal had lived for 13 years in a circus and was then transferred to an exotic felid sanctuary. According to the sanctuary veterinarian, the day before death the animal appeared depressed, regurgitated, and was ataxic, falling multiple times. An unknown dose of morphine was administered and the animal died 5 h later. At necropsy, both kidneys were surrounded by clotted blood in the retroperitoneum (Fig. 1a). The left testicle and spermatic cord were found intra-abdominal (cryptorchid) and grossly enlarged (30 × 9 cm) (Fig. 1a, b) compared to the right testicle (10 × 1.5 cm) (Fig. 1c), which was found in the scrotum. The enlarged left testicle was semi-hard with multiple yellow nodules from 0.5 to 6 cm in diameter involving the epididymis, pampiniform plexus, and deferent duct (Fig. 1b). On section, the masses were white to grey heterogeneous and vascular. Furthermore, a thrombus of 5 × 1.5 cm was found at the opening of the renal veins into the caudal vena cava (Fig. 1d). The lungs and mediastinal lymph nodes exhibited multiple semi-soft white nodules from 1 to 5 cm in diameter, consistent with metastases.

Samples were fixed in 10% neutral buffered formalin, processed by routine methods, embedded in paraffin wax, sectioned at 4 µm and stained with haematoxylin and eosin (HE) and periodic acid Shiff reaction (PAS). Immunohistochemistry against inhibin-α, Ki-67, human placental alkaline phosphatase (PLAP), cytokeratin AE1 + AE3, cKit, vimentin and S100 was carried out. Heat induced epitope retrieval was performed at 120 °C (pressure cooker) for 3 min in 10 mmol/L citrate buffer, pH 6.0. All primary antibodies were incubated overnight at 4 °C. For amplification and detection, the horseradish peroxidase polymer system (Hidef detection^®^, Cell Marque, Rocklin, CA, USA) and the avidin–biotin complex (Vectastain^®^, Vector Laboratories, Burlingame, CA, USA; for PLAP) were employed with 3,3′-diaminobenzidine as the chromogen and Harris’s Haematoxylin for counterstaining. Antibodies were tested with an internal control (cells expected to display positivity in lion tissues), as well as human and/or another felid (tiger) tissues as positive controls (Table 1).

**Table 1:** Immunohistochemistry panel: specificity, positive control, dilution and result for each antibody used for analyses

Primary antibody	Antibody specificity	Positive control	Dilution	Result
Vimentin	Clone V9 (Invitrogen^®^)	Internal (mesenchymal cells; fibroblasts lion)	1:2000/1500	(+)
S100	Polyclonal (Dako^®^)	Human intestine and tiger intestine (neural plexus)	1:5000	(+)
PLAP	Clone 8A9 (Dako^®^)	Human and tiger testicle (placental alkaline phosphatase in germ cells)	1:200	(−)
Inhibin	Clone Bc/R1 (Biocare^®^)	Human and tiger testicle (stromal and sex cord cells)	1:200	NI
Cytokeratin AE1 + AE3	Clone Isotyp IgG1 (Biocare^®^)	Human intestine and tiger intestine (epithelium)	1:1000	(−)
cKit/CD117	Clone YR145 (Cell Marque^®^)	Skin metastasis of human gastrointestinal stromal tumour and lion skin (cutaneous mastocytes)	1:500	(−)
Ki67	Clone MIB-1 (Dako^®^)	Human tonsil (tonsillar epithelium) and tiger intestine (epithelium)	1:100	NI

*NI* no-immunoreactivity

**Fig. 2:**
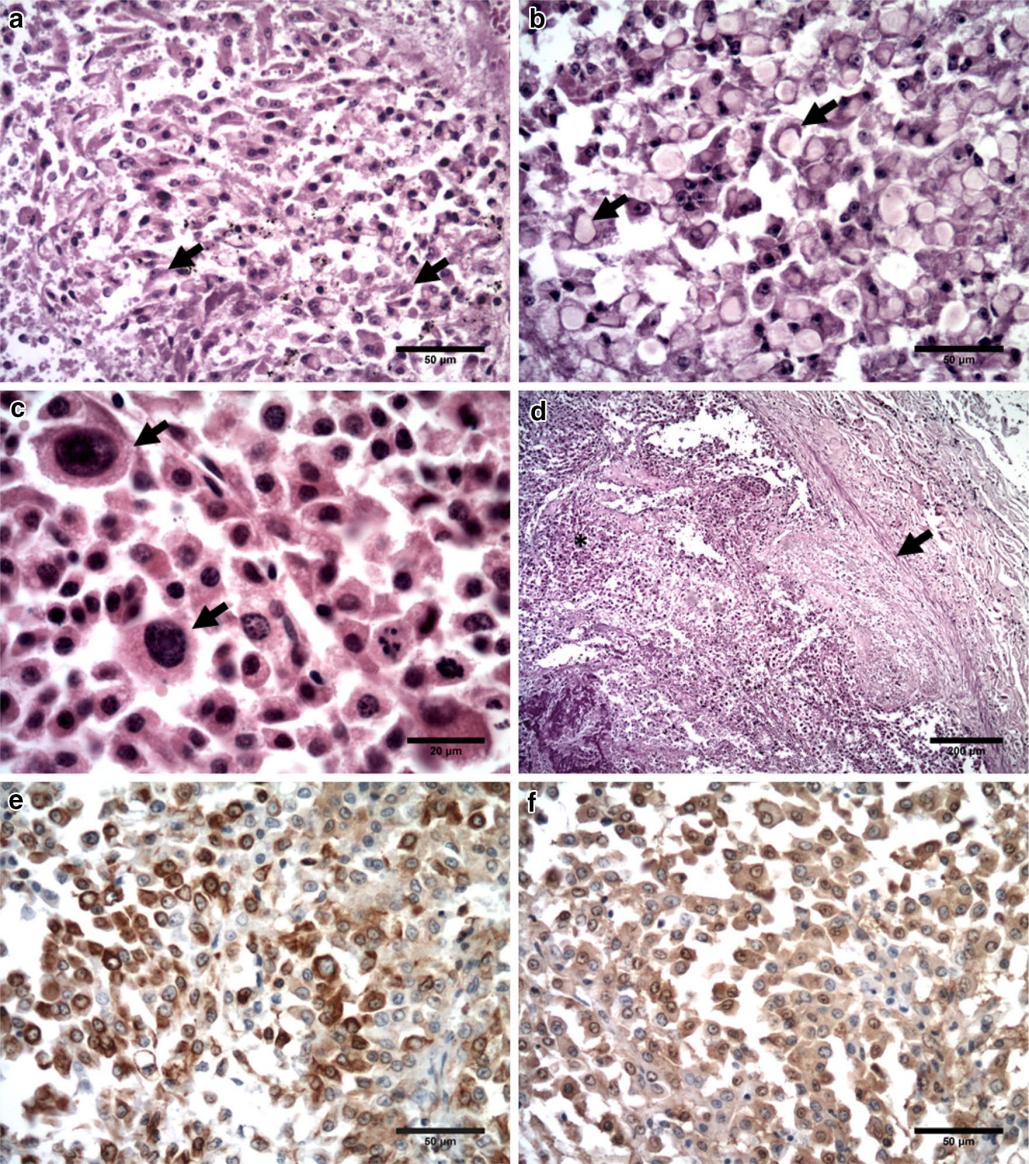
Microscopic images, neoplasm seen at the left testicle is poorly differentiated and pleomorphic composed of **a** round to polygonal cells with eosinophilic cytoplasm and scattered spindle cells (arrows), HE and **b** signet ring cells (arrows), HE. **c** Marked anisocytosis, anisokaryosis and karyomegaly (arrows) was noted in the neoplastic cells, HE. **d** Histological section of the neoplastic thrombus at a renal vein, the attachment is composed of fibrin (arrow). The lumen is occluded by a meshwork of tumour cells with fibrin (asterisk), HE. **e** Tumour cells shown strong cytoplasmic positive immunolabeling for vimentin. **f** Cytoplasm of neoplastic cells was positive for S100

Histologically, a highly cellular poorly differentiated pleomorphic neoplasm was present at the left testicle, composed predominantly of round to polygonal cells with scattered spindle cells (Fig. 2a) and “signet ring” cells (Fig. 2b). Neoplastic cells had moderate to abundant eosinophilic cytoplasm, were poorly delineated with round nuclei, fine chromatin and prominent nucleoli. Marked anisocytosis, anisokaryosis, karyomegaly (Fig. 2c), and a mitotic rate of 5 mitotic figures/high-power field (40 ×) was observed (Microscope Olympus, Model BX40). Multiple foci of mononuclear cell infiltration and mineralization were seen in the mass. Metastases had similar histologic features to the primary tumour. At the openings of the renal veins into the caudal vena cava, the thrombus showed multiple clusters of neoplastic cells attached to the luminal surface, surrounded by fibrin, erythrocytes and leukocytes (Fig. 2d). The right testicle was atrophied with no evidence of active spermatogenesis. Other diagnoses included multiple peribiliary cysts and proliferative glomerulonephritis with focally extensive erosive pyelitis. Neoplastic cells were PAS-negative, and presented strong positive cytoplasmic immunolabeling for vimentin (Fig. 2e) and S100 (Fig. 2f) immunohistochemistry. Antibody specificity, dilution, positive controls and immunohistochemistry results are displayed in Table 1.

## Discussion and conclusions

Considering the gross, microscopic and immunohistochemical findings, the neoplasm was diagnosed as a poorly differentiated pleomorphic sex cord–stromal tumour [12]. This classification has only been used in human medicine with support from immunohistochemistry with regard to differential diagnoses [13]. PLAP is a widely used antibody for seminoma diagnosis in humans and in domestic dogs. Classical seminoma is the predominant form in humans, expresses the germ cell markers PLAP and cKit, and is PAS-positive. The spermatocytic seminoma is derived from more differentiated cells and is the predominant form of seminoma in dogs [14–16]. The spermatocytic seminoma is PAS and vimentin negative and does not, or only focally, express PLAP and cKit in humans [17, 18]. cKit also has shown marked positivity in spermatocytic seminoma in humans [19]. Seminomas are mostly negative for cytokeratin AE1/AE3 [17, 20, 21] and S-100 [22], while Sertoli and Leydig cell tumours have a variable response for cytokeratin AE1/AE3 [23].

Because the histological and immunohistochemical features excluded the germ cell component, a poorly differentiated Leydig/Sertoli or sex cord–stromal neoplasm was considered. Occasional tumours of the testicles are considered in the sex cord–stromal tumour category, but as they do not fit into more specific categories, they are considered unclassified [24]. Some sex cord–stromal tumours are cytokeratin negative and label positive for S100 [22, 25], including those unclassified [12] and comprised predominantly of spindle cells [13]. Inhibin is a sensitive marker for sex cord–stromal tumours in humans [23] and Ki-67 is an important marker of proliferation, used for evaluating the prognosis of testicular neoplasms in several species such as dogs [26]. Inhibin and Ki-67 showed no immunoreactivity in the lion tissues in this study, either due to a lack of cross reactivity between the antibodies clones Bc/R1 (Inhibin) and MIB-1 (Ki-67) and the equivalent protein in lion´s tissues, or by a decrease/impairment of immunorecognition by processing conditions: e.g., xylene and/or paraffin infiltration, 10% neutral buffered formalin along with fixation time progression [27].

Sex cord–stromal tumours; Sertoli or Leydig cells tumours comprise a small percentage (~ 4%) of testicular neoplasms in men [12]. Unclassified sex cord–stromal tumours of the testicles are extremely rare in men [12] and should be considered to have a malignant potential [24]. Old age and cryptorchidism are important contributing factors to the development of testicular neoplasms, especially in dogs [11]. Sertoli cell tumours are the most common, especially when testicles are retained in the abdomen. Seminomas, the second most common neoplasm occurs mostly in inguinal retained testicles [11]. Cryptorchidism plays a role in the development of interstitial cell tumours in stallions and probably also in cats [11, 28]. Testicular tumour is 3–5% more likely in cases of cryptorchidism in humans [29]. Cryptorchidism is one of the most common abnormalities of the male reproductive system in humans and the most common in domestic cats [11]. Bilateral and multiple testicular tumours are relatively common, but metastases are rare [11]. Neoplasia in a retained testicle may be identified late in development, and they may become large and metastasize widely [11]. Sex cord stromal tumour, such as Sertoli cell tumour may infiltrate tissues adjacent to the testis, invade local vasculature and colonize adjacent lymph nodes and internal organs [28].

Thrombosis and haemorrhage are two major complications reported in cancer patients [30]. Thrombogenesis is based on Virchow’s triad: aberrant blood flow, loss of vascular integrity and altered blood components [31]. Neoplastic cells can activate the clotting system directly generating thrombin or indirectly by stimulating mononuclear cells to synthesize and express various pro-coagulants or by injuring endothelial cells and increasing coagulability [32]. Among humans with testicular tumours, those with germ cell tumours are at higher risk of thromboembolic events than those with non-germ cell tumours [33]. In germ cell tumours, metastases in the retro-peritoneum may invade or obstruct adjacent structures including the inferior vena cava [34, 35]. Involvement of the inferior vena cava in human germ cell tumours is uncommon [36]. In men, sex cord stromal tumour may mimic germ cell tumour and is occasionally aggressive [12].

The large size of the tumour in the lion, metastasis to the lung and mediastinal lymph nodes and thrombosis at the opening of the renal veins into the caudal vena cava may explain the behaviour and course of the neoplasm. In lions, a wide variant of carcinomas have been described to have metastasized [3, 37–40]. Pathological findings reported in other cases of testicular tumours in wild felids have included epistaxis [10] and pulmonary thrombosis [6], but this is the first report of a caudal vena cava thrombotic episode.

A metastatic sex cord–stromal tumour has not been previously reported in non-domestic felids. The severe perirenal retroperitoneal haemorrhage caused hypovolemic shock and is assumed to have been the cause of death of the lion.
